# Modulation of Macrophage Function by Bioactive Wound Dressings with an Emphasis on Extracellular Matrix-Based Scaffolds and Nanofibrous Composites

**DOI:** 10.3390/pharmaceutics15030794

**Published:** 2023-02-28

**Authors:** Tao He, Yuzhen Xiao, Zhijun Guo, Yifeng Shi, Qiuwen Tan, Yizhou Huang, Huiqi Xie

**Affiliations:** 1 Laboratory of Stem Cell and Tissue Engineering, Orthopedic Research Institute, State Key Laboratory of Biotherapy and Cancer Center, West China Hospital, Sichuan University and Collaborative Innovation Center of Biotherapy, Chengdu 610041, China; 2 Department of Breast Surgery, Clinical Center for Breast, West China Hospital, Sichuan University, Chengdu 610041, China; 3 School of Materials Science and Engineering, Southeast University, Nanjing 211189, China; 4 Department of Neurosurgery, West China Hospital, Sichuan University, Chengdu 610041, China

**Keywords:** bioactive materials, macrophage polarization, chronic wounds, wound dressing, extracellular matrix scaffolds, nanofibrous composites

## Abstract

Bioactive wound dressings that are capable of regulating the local wound microenvironment have attracted a very large interest in the field of regenerative medicine. Macrophages have many critical roles in normal wound healing, and the dysfunction of macrophages significantly contributes to impaired or non-healing skin wounds. Regulation of macrophage polarization towards an M2 phenotype provides a feasible strategy to enhance chronic wound healing, mainly by promoting the transition of chronic inflammation to the proliferation phase of wound healing, upregulating the level of anti-inflammatory cytokines around the wound area, and stimulating wound angiogenesis and re-epithelialization. Based on this, modulation of macrophage functions by the rational design of bioactive scaffolds has emerged as a promising way to accelerate delayed wound healing. This review outlines current strategies to regulate the response of macrophages using bioactive materials, with an emphasis on extracellular matrix-based scaffolds and nanofibrous composites.

## 1. Introduction

The skin is the largest organ in the human body and it protects internal organs against damage from external environments. In clinical practice, skin wounds caused by burns, trauma, surgical incisions, and chronic diseases such as diabetes and limb ischemia are very common [1]. It is well-known that normal skin wound healing is a sophisticated yet highly ordered process requiring proper interactions among repair cells, cytokines, and growth factors and can be broadly divided into four overlapping phases, including the hemostasis, inflammation, proliferation, and remodeling stages [2,3]. Notably, a variety of pathological factors, including but not limited to ischemia, chronic infection, and the malnutrition of patients, can contribute significantly to impaired or non-healing wounds because of the impedance of one or more stages of normal wound healing [1,2,3,4].

A bioactive wound dressing is adjunct with a biological activity that is applied to a wound to promote healing. Due to the progress of materials science and advanced manufacturing technology, a variety of bioactive scaffolds have been prepared that can not only provide physical protection for wounds (such as the maintenance of a stable moist environment in the wound bed), but also serve as a powerful tool to accelerate wound healing and boost skin tissue regeneration. In general, an ideal bioactive wound dressing should possess the following characteristics: (1) it can prevent the wounds from pathogenic bacteria infection and detrimental mechanical stimulation, improve the repair behaviors of cells, and regulate the microenvironment of wounds [5]; (2) it is biocompatible, breathable, waterproof, and dustproof, and should be capable of retaining water and absorbing exudates [6]; (3) it is easy to apply to and remove from the wounds without obvious tissue damage; (4) the degradation rate of wound dressings should match well with the rate of tissue regeneration [7]; and (5) the spatial structure of wound dressings should allow maximum cell interactions, provide sufficient space for tissue growth, and facilitate oxygen and nutrient transport. To meet these requirements, multi-functional bioactive wound dressings [8,9,10] are emerging as a hot research topic, typically designed based on the merits of natural and synthetic materials and the optimization of scaffolding methods.

With an increasing understanding of the mechanisms underlying biomaterial-mediated skin wound healing, it is generally considered that immune cells within the wound bed have critical roles in orchestrating the healing process [11]. Particularly, macrophages are the main effector cells regulated by biomaterials to improve the healing process [12]. For instance, inducing macrophage polarization towards an M2 phenotype (typically defined as both F4/80 and CD206 positive cells) by bioactive wound dressings—which enhance the secretion of anti-inflammatory cytokines and promote the transition of inflammation stage to the proliferation phase of wound healing—provides a feasible strategy to enhance chronic wound healing [13,14]. In this review, the critical roles of macrophages in normal skin wound healing and their dysfunction in impaired or chronic wounds are briefly introduced. Current strategies to regulate the response of macrophages with bioactive wound dressings are summarized, with an emphasis on extracellular matrix-based scaffolds and nanofibrous composites.

## 2. Roles of Macrophage in Normal Skin Wound Healing

Normal wound healing on the skin is a well-orchestrated process involving the hemostasis, inflammation, proliferation, and remodeling stages [15]. The presence and activation of macrophages are critical determinants for successful wound repair. According to their functions in wound healing, the activation of macrophages can be broadly classified into three categories: (1) the inflammatory macrophages, which are responsible for killing pathogens; (2) the resolving macrophages, which clear dead cells or cell debris and downregulate local inflammation; and (3) the tissue remodeling macrophages, which participate in tissue repair and remodeling [16]. Notably, the classification of macrophage activation does not represent distinct macrophage subpopulations but a continuum of macrophage activation that changes according to the local microenvironment [17].

At the inflammation stage of wound healing, cytokines and chemokines released by injured cells successively recruit neutrophils and monocytes to the wounds [18]. The recruited cells, together with tissue-resident immune cells, secrete a plethora of pro-inflammatory factors, such as interleukin-6 (IL-6) and granulocyte-monocyte colony-stimulating factor (GM-CSF), to recruit additional neutrophils and monocytes. With the induction of GM-CSF, M1 macrophages are transformed from the recruited monocytes and resident macrophages and release more pro-inflammatory cytokines in the wound area [19]. Cooperating with neutrophils, M1 macrophages clear damaged and dead cells, cell debris, foreign bodies, and pathogens in the wound bed [20].

At the proliferation stage of wound healing, M1 macrophages trigger the apoptosis and phagocytose of neutrophils [21]. The efferocytosis triggers the phenotypic switch from an M1 “pro-inflammatory” macrophage to an M2 “anti-inflammatory” phenotype [22]. M2 macrophages secrete anti-inflammatory factors, such as interleukin-10 (IL-10) and fibroblast growth factor (FGF) [23], which promote wound angiogenesis, granulation tissue formation, and re-epithelization [15,23,24]. At the remodeling stage of wound healing, M2 macrophages continuously secrete cytokines to promote the remodeling of granulation tissue with collagen-rich extracellular matrix and the maturation of new blood vessels [25].

## 3. Macrophage Dysfunction in Impaired or Chronic Wounds

Macrophage dysfunction, often characterized by a decreased ability to phagocytize pathogens or apoptotic cells, is an important contributor to impaired or chronic wounds. Notably, the phagocytosis of pathogens or apoptosis cells (i.e., efferocytosis) is key to activating the switch of the macrophage from the M1 pro-inflammatory phenotype to the M2 reparative phenotype [26]. Decreased efferocytosis leads to a reduced ratio of M2 macrophages while increasing the presence of M1 macrophages. Excessive M1 macrophages and persistent inflammation in the wounds inhibit the proliferation, differentiation, and migration of fibroblasts, endothelial cells, and keratinocytes, all of which are indispensable for wound healing. Additionally, M1 macrophages secrete massive metalloproteinases that inhibit new extracellular matrix (ECM) deposition and cleave resident ECM components [27]. Particularly, degraded ECM components can act as a chemoattractant to recruit additional macrophages, which further increases inflammation.

## 4. Strategies to Regulate Macrophage Function by Bioactive Scaffolds

### 4.1. Refinement of Scaffold Physical Properties

Physical properties of bioactive scaffolds, including the structure and morphology, have been considered an important factor influencing macrophage function, especially the polarization of macrophages [28].

#### 4.1.1. Pore Size

Several studies have shown that the pore size of scaffolds could modulate the biological behavior of macrophages [29,30,31]. Generally, scaffolds with a larger pore size tend to polarize macrophages toward an M2 pro-angiogenic phenotype while, smaller pores shift macrophages to an M1 pro-inflammatory phenotype [30,31,32,33,34]. Yin et al. showed that a genipin cross-linked collagen/chitosan scaffold with a larger pore size suppressed the expression of M1-related genes and cytokines (e.g., chemokine (C-C motif) ligand 2 (CCL2), interleukin-1β (IL-1β), interleukin-6 (IL-6), and vascular endothelial growth factor (VEGF)) and promoted the M1-to-M2 transition in macrophages [35]. Madden et al. demonstrated that hydrogel with a pore size of around 30–40 μm promoted the shift of macrophage phenotype toward an M2 state, resulting in increased vascularization and reduced fibrous encapsulation after implantation in vivo [29]. Similarly, another study showed that porous implants with uniformly interconnected 34 μm pores significantly reduced the foreign body response after subcutaneous implantation in mice, which was related to the shifting of macrophages to an M2 phenotype [36]. However, other research showed that titanium implants with larger pore sizes ranging from 40 to 160 μm did not show differences in cell ingrowth when implanted in rats [37], which suggests that a critical pore size of around 30 μm may be optimal to modulate macrophage function. It is noteworthy that these scaffolds are bioactive, so the pore size may not be the major factor to control macrophage behaviors. Furthermore, the pore size can be controlled by adding chemicals, while these chemicals themselves may affect macrophage function [38]. Therefore, the influence of scaffolds on the behavior of macrophages cannot be inferred simply by pore size.

#### 4.1.2. Fiber Diameter

Several studies have investigated the effect of fiber diameter on macrophage adhesion, proliferation, polarization, and the production of inflammatory cytokines [39,40]. For example, Garg et al. have investigated the influence of fiber diameter of electrospun mats on macrophage polarization in vitro, and they found that those fibers with a larger diameter favored the polarization of macrophages toward an M2 phenotype, increased the expression of M2 markers Arginase 1, VEGF, transforming growth factor-β1 (TGF-β1), and basic fibroblast growth factor (bFGF), and decreased the expression of M1 marker inducible nitric oxide synthase (iNOS) [32]. Similarly, another study reported that thicker-fiber scaffolds tended to induce macrophages to polarize into the M2 phenotype, while thinner-fiber scaffolds induced macrophages to polarize into the M1 phenotype [41]. However, some researchers hold different views. Horii et al. [42] implanted four types of non-woven poly-glycolic acid fabrics (with fiber diameters of 0.7, 0.9, 3.0, and 16.2 µm, respectively) into the rat dorsum. In the fabrics with diameters of 0.7 and 16.2 µm, more M1 macrophages were seen early around the surface of the fabric; by contrast, in those fabrics with diameters of 0.9 and 3 µm, M2 macrophages were predominantly observed throughout the fabric. Thus, they proposed that too thick and too thin fibers caused a continuous inflammatory response with massive M1 macrophages, whereas an appropriate fiber diameter inhibited the inflammation with plentiful M2 macrophages. Notably, it has been reported that the pore size of electrospun nanofibrous scaffolds increases with an increase in fiber diameter [43]. Thus, thicker fibers may cooperate with larger pore sizes to stimulate the polarization of macrophages.

#### 4.1.3. Stiffness

Recent studies have demonstrated that the stiffness of bioactive scaffolds can modulate macrophage function [44,45]. However, different conclusions exist in the literature regarding the influence of macrophage polarization. Most studies indicated that with increased stiffness in biological scaffolds, macrophages tend to polarize into the M1 phenotype, and the expression of pro-inflammatory cytokines, such as IL-1β, IL-6, and TNF-α, are upregulated [46,47,48]. For instance, Hsieh et al. observed that macrophages cultured on stiffer fibrin gels exhibited increased motility, enhanced inflammatory activation, and higher production of pro-inflammatory cytokines [49]. Similarly, He et al. reported that softer gels promote skin wound healing by enhancing the polarization of M2 macrophages and the upregulation of blood vessel formation [50]. In contrast, other research showed that stiffer scaffolds induce macrophages to polarize to an M2 phenotype [51,52]. Using the method of EDC-mediated crosslinking, Friedemann et al. successfully increased the stiffness of 3D collagen-based fibrillar matrices, and they found that a stiffer matrix resulted in an anti-inflammatory macrophage phenotype, even under inflammatory conditions (e.g., with the stimulation of GM-CSF) [53]. Additionally, Jiang et al. reported that when cultured with smaller and softer pores, macrophages exhibit a pro-inflammatory phenotype, whereas the anti-inflammatory phenotype is induced by larger and stiffer pores [54]. The inconsistent effect of matrix stiffness on macrophage behaviors, as described above, may be explained by large differences in the other properties of scaffolds, such as substrate composition, viscoelasticity, and porosity.

#### 4.1.4. Topography

Among the implant properties that can be manipulated, surface topography has attracted considerable attention since it can be readily modified. Several studies put forward that surface topography modulates macrophage behaviors by changing their cytoskeletal organization and cell adhesion [55,56]. After the culture of murine leukemic monocyte RAW 264.7 on a microrough surface, it was found that the surface microroughness activated the transcription of pro-inflammatory cytokine genes. However, when the microrough surface was modified to produce a more hydrophilic one with increased wettability and improved surface energy, the modified surface significantly downregulated the expression of pro-inflammatory cytokines (e.g., TNF-α, IL-1α, and IL-1β) [57]. Likewise, Hotchkiss et al. reported that hydrophilic rough surfaces induced macrophages to polarize to an anti-inflammatory M2-like state, which increased the expression of interleukin-4 (IL-4) and IL-10 [58]. In another study, it was observed that surfaces with micro- and nano-patterned grooves did not affect the inflammatory activation of macrophages but drove them toward an anti-inflammatory phenotype [59]. Although some studies support the conclusion that surface topography can modulate macrophage polarization through the change of cell morphology, others report a different result that topography-induced changes in the cell morphology do not link to a shift in macrophage polarization. For instance, compared with those cultured on planar surfaces, macrophages on concave and convex surfaces showed a different shape and size; however, no correlation was found with the polarization state [60].

### 4.2. Incorporation of Immunomodulatory Agents

#### 4.2.1. Cytokines

Cytokines are usually incorporated into bioactive scaffolds to enhance their immunomodulatory effect as common immunomodulatory agents [61,62,63,64,65,66]. IL-4 is one of the key cytokines that can skew macrophage polarization toward an M2 phenotype. Based on this, Bonito et al. designed an IL-4-functionalized supramolecular elastomer that can effectively promote the polarization of human monocyte-derived macrophages into an anti-inflammatory phenotype [63]. Similarly, Zhang et al. demonstrated that IL-4-loaded hydrogel beads promoted M2 macrophage polarization and increased the expression of TGF-β1 in vitro [64]. In another study by Xuan et al., an injectable bFGF-loaded chitosan-silver hydrogel was developed for the treatment of infected wounds. The incorporation of silver ions endorsed a controlled release of bFGF from the hydrogel. The in vivo results demonstrated that bFGF-loaded hydrogel significantly enhanced the repair of infectious wounds, which was partially ascribed to the promotion of M2 macrophage polarization and reduced inflammatory reactions [65]. Moreover, it is shown that controllable release of stromal cell-derived factor-1 alpha from a nanocomposite hydrogel system influenced the recruitment and polarization of macrophages in diabetic wounds, demonstrating primarily the anti-inflammatory phenotype of macrophages, and resulted in increased epidermal stratification, enhanced dermal angiogenesis, and faster wound closure [66].

#### 4.2.2. Chemical Compounds

Many chemical compounds with the ability to modulate macrophage functions have been incorporated into wound dressings [67,68,69,70,71,72]. For instance, different kinds of polyphenols (i.e., tannic acid, oligomeric proanthocyanidins, and epigallocatechin-3-O-gallate) have been added into hydrogels, and it is observed that the distribution of CD206, a typical marker of M2 macrophages, was more abundant in the polyphenol-loaded hydrogels after implantation at the full-thickness wounds of mice. Additionally, VEGF, one of the key cytokines secreted by M2 macrophages, presented a superior distribution in the polyphenol-loaded hydrogels as compared with hydrogels without polyphenols [69]. Neurotensin (NT), a neuropeptide that acts as an inflammatory modulator in wound healing, has been loaded on collagen dressings. NT-loaded collagen matrices were found to promote diabetic wound healing and significantly reduced the expression of inflammatory cytokines (TNF-α and IL-1β) [70]. Wu et al. reported that a prolonged release of taurine, an amino acid in mammal species, improved the healing of full-thickness skin wounds, which was related to the stimulation of M2 macrophage polarization and a decreased inflammatory response [71]. Similarly, artemisinin (ART), a sesquiterpene lactone compound extracted from *Artemisia annua* L., has received great interest because of its anti-inflammatory properties [73]. Using electrospinning technology, Peng et al. fabricated a polylactic acid glycolic acid/silk fibroin membrane loaded with artemisinin. The in vitro results showed a good, sustained release of artemisinin and favorable anti-inflammatory properties. In a full-thickness skin wound model, the artemisinin-loaded membranes shortened the period of inflammation and enhanced skin regeneration through the downregulation of pro-inflammatory cytokines, IL-1β and TNF-α [72].

### 4.3. Loading with Stem Cells or Their Derivates

#### 4.3.1. Stem Cells

In addition to immunomodulating agents, stem cells and their derivatives are hot agents to enhance the ability of biomaterials to regulate macrophage function. Mesenchymal stem cells (MSCs), with robust self-renewal and multidirectional differentiation abilities, are one of the most popular seed cells in the fields of tissue engineering and stem cell-based therapy [74]. MSCs can secrete immunomodulatory cytokines and growth factors that exert paracrine effects on immune cells to promote wound healing. To obtain a better spatial delivery of stem cells and to improve their retention in wound areas, different kinds of biomaterials have been fabricated to serve as versatile cell carriers. Particularly, MSC-loaded scaffolds have been reported to promote skin wound healing by modulating macrophage function [8,75]. Lin et al. [76] demonstrated that MSC-loaded fish scale scaffolds can effectively convert activated M1 macrophages into an M2 phenotype in the wounds and reduce inflammation around the flap to promote skin regeneration. When delivered to full-thickness wounds by collagen-based scaffolds, human dermal- and adipose tissue-derived MSCs showed accelerated wound healing speed and promoted wound repair with tissues more similar to normal skin. Notably, both MSCs induced macrophage polarization in the wounds, shifting from a pro-inflammatory (M1) to a pro-repair (M2) phenotype [77]. Moreover, in burn wounds, adipose tissue-derived MSCs in combination with injectable polysaccharide hydrogels have shown good healing outcomes. The ADSCs-laden hydrogels effectively reduced the inflammatory response, enhanced macrophage M2 polarization, and significantly accelerated wound healing [78].

#### 4.3.2. Stem Cells Derivates

Exosomes derived from adult stem cells, especially those from MSCs, are frequently incorporated into biomaterials for skin wound treatment. Xiao et al. demonstrated that exosomes isolated from adipose tissue MSCs are very beneficial for diabetic wound healing. The exosomes were loaded on a human acellular amniotic membrane, and the in vivo results showed that the exosome-loaded wound dressing accelerated wound healing by regulating inflammation, vascularization, and ECM deposition [79]. In addition to exosomes, small extracellular vesicles (sEVs) derived from MSCs also possess great therapeutic potential for skin wounds, mainly because of their ability to deliver a variety of bioactive factors. With an optimized release profile from micro/nanofiber scaffolds, sEVs derived from adipose MSCs have been reported to induce the polarization of macrophages toward a pro-healing M2 phenotype in vitro. They further improved diabetic wound healing in vivo by alleviating the inflammatory response [80].

## 5. Different Types of Bioactive Wound Dressings to Modulate Macrophage Function

### 5.1. ECM-Based Scaffolds

ECM is a multifunctional complex of biomacromolecules (mainly containing proteins and polysaccharides), which is first synthesized by body cells and then secreted into the extracellular space of tissues [81]. ECM has long been considered good at providing sufficient structural support and a beneficial microenvironment for the proliferation and differentiation of wound repair cells [82]. As a biodegradable material containing diverse bioactive molecules, ECM plays a fundamental role in immunomodulation, angiogenesis, and collagen secretion [83]. Based on these advantages, ECM-based scaffolds are regarded as a good option for skin wound healing.

To mimic the biological function of natural ECM, decellularized ECM scaffolds are excellent candidates due to the advantages of greatly conserving the structure and composition of native ECM [84]. In addition to a similar physical structure, decellularized ECM scaffolds also contain the biochemical components of natural tissues and thus possess the capability of modulating the recruitment, migration, and polarization of macrophages [85]. Actually, several studies demonstrate that decellularized ECM scaffolds derived from different tissue sources exert different effects on the biological behavior of macrophages [86,87,88]. For instance, it is reported that decellularized ECM scaffolds derived from the urinary bladder, esophagus, colon, and small intestinal mucosa can polarize macrophages toward an M2 phenotype, whereas dermal decellularized ECM scaffolds induce macrophages to represent an M1 phenotype [86,87]. However, decellularized ECM scaffolds derived from skeletal muscle have no effect on macrophage polarization [86]. As for brain-decellularized ECM scaffolds, the type of macrophage polarization is controversial [86,87].

In fact, an increasing number of studies have focused on the effect of ECM-based scaffolds on macrophage function, with the primary aim of promoting skin wound healing (Table 1) [89,90,91]. As a dense tissue with abundant ECM and a sparsely cellularized structure, the pericardium represents a suitable candidate for producing decellularized ECM scaffolds [92]. El Masry et al. reported that stabilized, acellular, pericardial collagen matrix (sPCM), which was made from the equine pericardium, was effective in resolving robust wound inflammation and promoting the healing rate and quality. Along with faster and better wound healing in vivo, this wound dressing induced the expression of antimicrobial proteins in keratinocytes and inhibited the adhesion of bacteria in vitro [93]. Similarly, ECM hydrogels produced from pericardium also show good immunomodulatory, angiogenic, and wound healing potential. It is demonstrated that bovine pericardium ECM hydrogel tends to skew M0 macrophages towards an M2 phenotype in vitro, showing a decreased expression of M1 phenotype markers (CD80 and CD86) and an upregulated expression of M2 phenotype markers (CD163 and CD206) when cultured with macrophages. Furthermore, in a murine skin wound model, the hydrogel was found to promote wound healing by inducing M2 macrophage polarization, enhancing fibroblast migration, and stimulating angiogenesis [94].

Recently, we have fabricated a bioactive wound dressing using human ADSCs as seed cells and porcine small intestinal submucosa (SIS), an ECM scaffold, as the cell delivery vehicle. When applied in rat diabetic wounds, the wound dressing promoted macrophage infiltration at the early stage of wound healing and reduced the infiltration at later stages. Particularly, the wound dressing modulated macrophage polarization into an M2 phenotype and enhanced wound healing through stimulating angiogenesis, re-epithelialization, and skin appendage regeneration [75]. In addition to SIS, other tissue-derived ECM scaffolds, such as acellular dermal matrix (ADM) and acellular amniotic membrane, have been commercialized and applied in the clinic. More advanced wound dressings based on these tissue-derived ECM scaffolds are in rapid development, such as photocrosslinkable hydrogels [95].

Apart from ECM scaffolds derived from human or animal tissues, cell-derived ECM has been explored for skin wound healing. The composition of cell-derived ECM can be tailored by choosing a suitable cell source, cell culture condition, and decellularization method, making it possible to engineer a pro-healing microenvironment. Savitri et al. reported that ECM scaffolds prepared from different cell sources showed substantially different intrinsic properties, such as fibrous matrix, total protein, and biochemical factors. Furthermore, in a full-thickness mouse skin wound model, they observed that ECM derived from human lung fibroblasts or umbilical cord-blood MSCs can effectively promote wound healing, showing a large proportion of M2 macrophages, high recruitment of myofibroblasts, and significant recovery of skin appendages [96]. Notably, from the view of clinical translation, a scale-up production of cell-derived ECM awaits further studies.

### 5.2. Nanofibrous Composites

Because they have many desirable properties for skin wound healing, nanofibrous composites have received significant attention in the past few decades [97,98,99,100,101]. Nanofibers can be designed to possess a random or aligned structure, and the fiber diameter can be adjusted to closely match that of collagen fibrils in the natural skin ECM (i.e., the range of 100–200 nm). With a high microporosity and permeability, nanofibers are ideal for exudate absorption and the diffusion of oxygen and nutrients. Furthermore, a high surface area of nanofibers is beneficial for cell adhesion and proliferation.

To enhance the wound therapeutic efficacy of nanofibrous composites, a variety of bioactive factors [80,102,103,104,105,106,107,108,109], such as immunomodulators [103], stem cells and their derivatives (e.g., exosomes, conditioned medium, small extracellular vesicles (sEVs), etc.) [80,106], have been incorporated into scaffolds (Table 2). For instance, ursolic acid (UA) extracted from Chinese herbal plants has been encapsulated into a nanofibrous wound dressing. The UA-loaded nanofibers significantly reduced the M1 phenotypic polarization of macrophages and effectively restored the M2 polarization in vitro. In a diabetic wound model, this scaffold was found to improve wound closure, re-epithelialization, revascularization, and hair follicle regeneration [99]. Similarly, anemoside B4, an anti-inflammatory ingredient of the Chinese medicine *Pulsatilla*, has been loaded into an electrospun nanofibrous wound dressing, which significantly suppressed the lipopolysaccharide-stimulated differentiation of pro-inflammatory macrophages in vitro and accelerated diabetic wound healing in vivo [103]. In addition, it is reported that, in both normal and diabetic wounds, reduced graphene oxide-loaded isabgol nanocomposite scaffolds can reduce the inflammatory response, stimulate collagen synthesis, and enhance wound contraction and re-epithelization [109]. MSCs and their derivates have been proven to promote skin tissue repair [1,110,111]. Li et al. fabricated a novel micro/nanofiber wound dressing to release sEVs derived from MSCs. When cultured with macrophages in vitro, the sEVs-loaded dressing inhibited the expression of pro-inflammatory cytokines (IL-1β and TNF-α) and upregulated anti-inflammatory cytokines (IL-10, agr-1, and CD206). In a diabetic wound model, the dressing was found to promote wound healing by alleviating inflammatory responses and stimulating fibroblast proliferation, collagen deposition, and wound angiogenesis [80].

### 5.3. Other Bioactive Wound Dressings

#### 5.3.1. Nanoparticle Loaded Scaffolds

Nanoparticles (NPs), with diameters ranging roughly from one to a few hundred nanometers, have been proven to be capable of inducing favorable biological responses to promote skin wound healing. For instance, silver NPs possess antimicrobial activity for infected wounds [112]; gold NPs possess the antioxidant property for diabetic wound treatment [113]; and calcium NPs can regulate the migration of keratinocytes [114]. Notably, under an appropriate magnetic field, ligand-bearing superparamagnetic iron oxide NPs were reported to be capable of manipulating the adhesion and polarization of macrophages in vitro and in vivo [12,115], and the tunable delivery system has great value for precise control of macrophage phenotype. To be specific, the magnetic field was applied at different oscillation frequencies to manipulate the frequency-dependent ligand nano-oscillation speeds of the ligand-bearing superparamagnetic iron oxide NPs, which further modulates the adhesion and polarization of macrophages. For instance, Kang et al. reported that a low oscillation frequency of the magnetic field and M2-inducing cytokines synergistically stimulated the M2 polarization of macrophages, whereas a high oscillation frequency suppressed the adhesion of macrophages and promoted their M1 polarization [115].

With a suitable encapsulation efficiency, NPs are usually introduced into hydrogels via physical entrapment, chemical cross-linking, or other methods to further enhance the wound repair potential of scaffolds [107,116]. Puertas-Bartolomé et al. fabricate a catechol-bearing NPs hydrogel and demonstrate that it possesses excellent wound healing capacities, including the regulation of ROS production, an anti-inflammatory response, and the upregulation of VEGF expression [117]. Similarly, Ni_4_Cu_2_ hollow nanospheres, applied in combination with F127 hydrogel, were found to effectively promote wound healing, epithelial regeneration, and the formation of hair follicles in mice [118]. Moreover, Zhu et al. prepared an injectable hydrogel containing substance P-loaded NPs, which showed excellent wound-healing efficacy by promoting an early inflammatory response and the subsequent M2 macrophage polarization in full-thickness wounds [119]. Additionally, IL-10 released from collagen-silica nanocomposites was also reported to decrease TNF-α and IL-1β gene expression and favor the expression of wound-healing cytokines [120].

#### 5.3.2. Hydrogel Wound Dressings

A great variety of hydrogels have been used in skin regeneration due to the advantages of modulating macrophage behaviors, accelerating blood vessel formation, and promoting collagen deposition [121,122]. Lu et al. [123] reported a gelatin-fucoidan-tannic acid hydrogel wound dressing, which was found to significantly inhibit the expression of macrophage signal transducer and activator of transcription (STAT) 1 (a signaling pathway for M1 macrophage polarization) at rat full-thickness skin wounds, and obviously increase the expression of STAT 6 (a signaling pathway for M2 macrophage polarization). In addition, the expression of pro-inflammatory factors around the wound, such as IL-6, IL-1β, and TNF-α, was significantly decreased, and the level of anti-inflammatory factors, such as IL-10, arg-1, and TGF-β, was significantly increased. All of this suggests that hydrogel can modulate macrophage polarization and alleviate inflammatory responses to promote skin wound healing.

Similarly, Sharma et al. developed a hydrogel scaffold using polyelectrolyte complexation (PEC) between the cationic polysaccharide chitosan (CH) and an anionic glycosaminoglycan chondroitin sulfate (CS). The CH-CS PEC hydrogel increased the phagocytic ability of macrophages in vitro, showing a low expression level of TNF-α and a high production of IL-10. Furthermore, in an infected wound model, the hydrogel enhanced wound closure with reduced inflammation and increased angiogenesis [124]. In a bacteria-infected wound model, a Co^2+^-Ca^2+^/Gauze/sodium alginate composite wound dressing was reported to recruit macrophages around the wounds and increase the level of CD206, a typical marker of M2 macrophages, to promote skin wound healing [125]. Furthermore, hydrogels can serve as carriers for stem cells or cell derivatives (such as adipose-derived MSCs [78], platelet-rich plasma [122], and platelet-derived extracellular vesicles [126]), and a synergistic effect of hydrogels and the loading materials can further enhance M2 macrophage polarization to accelerate skin wound healing [122,126].

## 6. Future Perspective

Skin wound healing is a continuous process in which the plasticity of macrophages, more specifically, the different phenotype of macrophages after polarization, plays a key role. At the early stage of wound healing, macrophages mainly polarize into the pro-inflammatory M1 phenotype, which promotes inflammation, swallows pathogens, and eliminates apoptotic and damaged cells. At the later stages of wound healing, macrophages predominately polarize into the anti-inflammatory M2 phenotype, which secretes a plethora of pro-healing factors to enhance epidermal regeneration, wound angiogenesis, and collagen deposition. In poorly healed or unhealed wounds, a prolonged inflammatory period, excessive inflammatory response, extensive infiltration of M1 macrophages, and a shortage of M2 macrophages are often observed in the clinic. Thus, the timely induction of macrophages to polarize into an M2 phenotype represents a reasonable therapeutic target for chronic wounds.

With an increased understanding of wound biology, different kinds of bioactive wound dressings have been reported in the literature that can effectively modulate the function of macrophages in wounds. However, most of these scaffolds just regulate the behavior of macrophages in a single way. They unidirectionally induce the polarization of macrophages toward an M2 phenotype. It is noteworthy that, at the early stage of wound healing, the polarization of macrophages toward an M2 phenotype seems to be opposite to the expected polarization of macrophages. Therefore, how can we manufacture bioactive scaffolds that are smart enough to play different roles in modulating macrophage function according to the stage of wound healing and the status of the local microenvironment? In other words, how do you achieve a continuous and ordered coordination of inflammatory and anti-inflammatory macrophages after the application of wound dressings? This is not only a major technical difficulty in developing bioactive wound dressings at present but also an important direction for the manufacture of biomaterials in the future. Undoubtedly, successful engineering of such wound dressings will be very helpful to prevent the occurrence of chronic wounds in patients with severe diseases such as diabetes and lower limb ischemia. Furthermore, although many studies have demonstrated that bioactive wound dressings can modulate macrophage function in vivo, the mechanism underlying scaffold-mediated polarization of macrophages has not yet been fully understood, which should be the focus of future studies.

## 7. Conclusions

The polarization of macrophages to fulfill proper functions is critical for successful skin wound healing. Researchers have recently been trying to improve chronic wound healing by engineering biomaterials that modulate macrophage function. A variety of bioactive wound dressings, particularly ECM-based scaffolds and nanofibrous composites, have been successfully fabricated with outstanding wound-healing potential. Through the induction of macrophage polarization towards an M2 phenotype, these wound dressings are verified to stimulate the secretion of anti-inflammatory cytokines and consequently enhance wound contraction, re-epithelialization, angiogenesis, and ECM deposition. Several strategies have been developed to improve the macrophage modulation ability of wound dressings, including the refinement of scaffold physical properties (pore size, fiber diameter, stiffness, and topography), the incorporation of immunomodulatory agents, and the loading with stem cells or their derivates, all of which showed great value for next-generation wound management. Despite much progress in this field, few clinical trials have been carried out, and future studies should be focused on their clinical translation potential.

## Figures and Tables

**Table 1:** ECM-based scaffolds capable of modulating macrophage function to promote skin wound healing.

Scaffold	Tissue/Cell Source	Wound Healing Outcomes	Macrophage Behaviors	Ref.
sPCM	Equine pericardium	Accelerated wound closure; increased collagen deposition and maturation.	Polarized into M2 phenotype; elevated the levels of IL-10, Arginase-1 and VEGF; and reduced the expression of IL-1β and TNF-α.	[93]
Pericardium dECM hydrogel	Bovine pericardium	Enhanced wound healing.	Polarized into M2 phenotype; reduced the expression of CD80/CD86; and increased the expression of CD163 and CD206.	[94]
SIS + ADSCs	SIS	Enhanced wound angiogenesis, re-epithelization, and skin appendage regeneration.	Promoted macrophage infiltration at the early stage of wound healing; reduced macrophage infiltration at later stages of wound healing; and raised the M2:M1 macrophage ratio.	[75]
DTP4 hydrogel	Decellularized dermal tissue	Raised wound closure ratio; reduced inflammatory response; and promoted re-epithelization, hair follicles regeneration, and collagen maturation.	Shifted to the healing phase quickly.	[95]
hFDM; UMDM	Human lung fibroblasts; umbilical cord-blood MSCs	Thinner epidermal layer; significant recovery of skin appendage; better neovascularization; and higher recruitment of myofibroblasts.	A large number of M2 macrophage; most of CD206^+^ cells in the dermis region; and ECM Hydrogel reserved more CD206^+^ cells than the other ones.	[96]

ADSCs—adipose tissue-derived stem cells; dECM—decellularized extracellular matrix; ECM—extracellular matrix; hFDM—human lung fibroblast-derived matrix; MSCs—mesenchymal stem cells; sPCM—stabilized, acellular, equine pericardial collagen matrix; SIS—small intestinal submucosa; and UMDM–umbilical cord-blood mesenchymal stem cells derived matrix.

**Table 2:** Nanofibrous composites to modulate macrophage function for wound healing.

Nanofibrous Composites	Materials Combined with Nanofibers	Biological Function	Ref.
CS-PVA-UA	UA	Reduced M1 macrophage polarization and restored M2 polarization; improved wound closure rate; promoted re-epithelization, revascularization, collagen deposition, and remodeling; and stimulated hair follicles regeneration.	[99]
sEVs@DSPE-PLLA	adipose MSC-derived sEVs	Inhibited the expression of IL-1β and TNF-α; upregulated the expression of IL-10, Arginase 1, and CD206; stimulated fibroblast proliferation; and promoted collagen deposition and angiogenesis.	[80]
CS-PVA-ANE	ANE	Suppressed LPS-stimulated M1 macrophage polarization; reduced ROS production; decreased the level of inflammatory cytokines; enhanced wound closure; accelerated wound angiogenesis; and promoted wound re-epithelization and collagen deposition.	[103]
Isab + rGO	rGO	Stimulated collagen synthesis, collagen crosslinking, and wound contraction; reduced re-epithelization time; and reduced the presentence of CD68 positive cells.	[109]
PFKU nanofibrous membrane	DOXH	Promoted the migration of endothelial cells; promoted macrophage polarization into the M2 phenotype; downregulated the level of ROS and inflammatory factors; Upregulated the ratio of M2 macrophage; and promoted collagen deposition, revascularization, and re-epithelization.	[107]
PVP-I/PCL-poly-L-lysine nanofibers	PVP-I	Reduced the level of pro-inflammatory cytokines (TNF-α and IL-1β); promoted cell adhesion; and antimicrobial property.	[105]
PCL/gelatin nanofibers	NAGEL	Promoted the adhesion, proliferation, and migration of endothelial cells and keratinocytes; induced angiogenesis, collagen deposition, and re-epithelization of wounds; inhibited the inflammatory reactions; and activated EMT and EndMT pathways.	[108]

ANE—anemoside; CS—chitosan; DOXH—doxycycline hydrochloride; EMT—epithelial to mesenchymal transition; EndMT—endothelial to mesenchymal transition; Isab + rGO—reduced graphene oxide loaded isabgol nanocomposite scaffold; NAGEL— nagelschmidtite; PCL—polycaprolactone; PFKU—polyurethane; PVA—polyvinyl alcohol; PVP-I—poly(vinyl pyrrolidone)-iodine; rGO—reduced graphene oxide; ROS—reactive oxygen species; sEVs—small extracellular vesicles; and UA—ursolic acid.

## Data Availability

Not applicable.

## Abbreviation

AAM, acellular amniotic membrane; ADM, acellular dermal matrix; ADSCs, adipose derived stem cells; ANE, anemoside; ART, Artemisinin; CCL2, chemokine (C-C motif) ligand 2; CH, chitosan; CS, chondroitin sulfate; dECM, decellularized extracellular matrix; DOXH, doxycycline hydrochloride; ECM, extracellular matrix; EMT, epithelial to mesenchymal transition; EndMT, endothelial to mesenchymal transition; FGF, fibroblast growth factor; GM-CSF, granulocyte-monocyte colony-stimulating factor; hFDM, human lung fibroblast derived matrix; IL-10, interleukin-10; IL-1α, interleukin-1α; IL-1β, interleukin-1β; IL-4, interleukin-4; IL-6, interleukin-6; iNOS, inducible nitric oxide synthase; Isab + rGO, reduced graphene oxide loaded isabgol nanocomposite scaffold; LPS, lipopolysaccharide; MSCs, mesenchymal stem cells; NAGEL, nagelschmidtite; NO, nitric oxide; NPs, nanoparticles; NT, neurotensin; PCL, polycaprolactone; PEC, polyelectrolyte complexation; PFKU, polyurethane; PVA, polyvinyl alcohol; PVP-I, poly(vinyl pyrrolidone)-iodine; rGO, reduced graphene oxide; ROS, reactive oxygen species; sEVs, small extracellular vesicles; SIS, small intestinal submucosa; sPCM, stabilized, acellular, equine pericardial collagen matrix; STAT, signal transducer and activator of transcription; TGF-β1, transforming growth factor β1; TNF-α, tumor necrosis factor-α; UA, ursolic acid; UMDM, umbilical cord-blood mesenchymal stem cells derived matrix; VEGF, vascular endothelial growth factor.
